# Paradoxical choice and the reinforcing value of information

**DOI:** 10.1007/s10071-022-01698-2

**Published:** 2022-10-28

**Authors:** Victor Ajuwon, Andrés Ojeda, Robin A. Murphy, Tiago Monteiro, Alex Kacelnik

**Affiliations:** 1 Department of Biology, University of Oxford, Oxford, UK; 2 Department of Experimental Psychology, University of Oxford, Oxford, UK; 3 Domestication Lab, Department of Interdisciplinary Life Sciences, Konrad Lorenz Institute of Ethology, University of Veterinary Medicine Vienna, Vienna, Austria

**Keywords:** Conditioned reinforcement, Non-instrumental information, Paradoxical choice, Suboptimal choice, Stimulus salience, Rat

## Abstract

Signals that reduce uncertainty can be valuable because well-informed decision-makers can better align their preferences to opportunities. However, some birds and mammals display an appetite for informative signals that cannot be used to increase returns. We explore the role that reward-predictive stimuli have in fostering such preferences, aiming at distinguishing between two putative underlying mechanisms. The ‘information hypothesis’ proposes that reducing uncertainty is reinforcing per se*,* somewhat consistently with the concept of curiosity: a motivation to know in the absence of tractable extrinsic benefits. In contrast, the ‘conditioned reinforcement hypothesis’, an associative account, proposes asymmetries in secondarily acquired reinforcement: post-choice stimuli announcing forthcoming rewards (S^+^) reinforce responses more than stimuli signalling no rewards (S^−^) inhibit responses. In three treatments, rats faced two equally profitable options delivering food probabilistically after a fixed delay. In the informative option (*Info)*, food or no food was signalled immediately after choice, whereas in the non-informative option (*NoInf*o) outcomes were uncertain until the delay lapsed. Subjects preferred *Info* when (1) both outcomes were explicitly signalled by salient auditory cues, (2) only forthcoming food delivery was explicitly signalled, and (3) only the absence of forthcoming reward was explicitly signalled*.* Acquisition was slower in (3), when food was not explicitly signalled, showing that signals for positive outcomes have a greater influence on the development of preference than signals for negative ones. Our results are consistent with an elaborated conditioned reinforcement account, and with the conjecture that both uncertainty reduction and conditioned reinforcement jointly act to generate preference.

**Supplementary Information:**

The online version contains supplementary material available at 10.1007/s10071-022-01698-2.

## Introduction

Models of instrumental learning in animals, and of reinforcement learning in machines, argue that agents increase the frequency of actions that result in higher probability of desirable consequences, and reduce the frequency of actions with undesirable ones. Desirability and aversiveness have adaptive roots in animals (for a review see Staddon and Cerutti 2003), while in machines reinforcement criteria are built in by design (Sutton and Barto 2018). In animals, constrained essential commodities or other substantial beneficial outcomes are effective reinforcers, and it is not surprising that in laboratory studies signals for their occurrence or absence modulate animals’ lever pressing or key-pecking responses. However, a question that has recently seen a resurgence of interest in the psychological (Cunningham and Shahan 2018; Shahan and Cunningham 2015), neuroscientific/robotics (Gottlieb and Oudeyer 2018; van Lieshout et al. 2020), and computational (Dubey and Griffiths 2020) literatures is whether in addition to such commodities, information (reductions in uncertainty) modulates behaviour through the same processes that conventional rewards do, that is, whether information can act as a primary reinforcer.

In a world where uncertainty is pervasive, information is a valuable asset that can be used by decision-makers to enhance efficiency in activities such as foraging, mating or homing, to improve their performance (Behrens et al. 2007; Dall et al. 2005) and ultimately contribute to Darwinian fitness. In instrumental tasks, animals may seek information before making choices (Gottlieb et al. 2014) and this can improve the acquisition of commodities (Foley et al. 2017; Kobayashi and Hsu 2019). In such contexts, the adaptive and reinforcing values of information-seeking derive from its ability to increment a well-defined benefit, in which case traditional functional and mechanistic accounts are aligned.

However, what seems paradoxical with reference to normative models of reward-maximisation across fields such as microeconomics and foraging theory (e.g., Mas-Colell et al. 1995; Stephens and Krebs 1986), and classical models of reinforcement learning (Rescorla and Wagner 1972), is that animals show preferences for informative signals in cases where the signals have no potential instrumental use—that is, they seek out information ‘for its own sake’, are ‘uncertainty averse’, or are ‘curious’ (Bromberg-Martin and Hikosaka 2009; Cervera et al. 2020; Kidd and Hayden 2015). The idea that animals value information irrespective of its ability to increase rewards was postulated to explain the experimental phenomenon of the ‘observing response’, first explored by Wyckoff (1951, *unpublished thesis;* see Wyckoff 1969). In this paradigm, subjects can resolve uncertainty about forthcoming contingencies by performing a response, though the information provided cannot be used to modify outcomes. Wyckoff presented pigeons with a white key and a mixture of two reinforcement schedules. In the rich schedule, pecking the key resulted in food delivery every 30 s, while in the poor schedule pecking at the key did not produce food. The system alternated periodically and unpredictably between both schedules. The critical aspect was the addition of a pedal such that if the animal stepped on it, then the white key turned red when the system was in its rich state and green during the poor periods. The pedal response informed the animal of the current state of the world but did not modify it. The pigeons readily acquired pedal pressing, which was appropriately labelled an ‘observing response’. Similar procedures have been conducted with variable delays to food (Bower et al. 1966) and aversive outcomes such as electric shocks (Lockard 1963). In all cases, animals acquire such observing responses; they choose to elicit signals that resolve uncertainty about probabilistic future outcomes, without altering these outcomes.

A number of theoretical hypotheses have been proposed to explain apparent information-seeking behaviour in the observing response paradigm (for a review see Dinsmoor 1983) and other protocols derived from it. One candidate mechanistic explanation, which we will call the ‘information’ or ‘uncertainty reduction’ hypothesis—informed by classical information theory (Shannon 1948)—suggests that animals find information itself intrinsically rewarding because it relieves uncertainty, which has negative hedonic valence (Berlyne 1960, 1957; Hendry 1969). According to this account, information (and by extension an event or stimulus that reduces uncertainty) acts as a primary reinforcer modulating behaviour. Functionally, this could evolve if information is often associated with substantive benefits in ecological contexts and is not too costly to acquire. This view is consistent with notions of ‘curiosity’ defined as a motivation to ‘know’ for the sake of it, or acquire information in the absence of instrumental incentives (Cervera et al. 2020; Gottlieb and Oudeyer 2018; Kidd and Hayden 2015). The idea that individuals value information has also recently been explored in humans. Bennet et al. (2016) suggested that information may be valued because it prevents temporally prolonged uncertainty, which is presumed to be aversive. Other investigators have proposed that information may derive its reinforcing value by enabling subjects to appetitively ‘savour’ good news about positive outcomes while waiting for those outcomes (Brydevall et al. 2018; Iigaya et al. 2016).

An alternative mechanistic explanation, which we refer to as the ‘conditioned reinforcement hypothesis’ (Bower et al. 1966; Prokasy 1956; Wyckoff 1959), prescinds of attributing reinforcing properties to information per se*.* It instead proposes that the signal for ‘good news’ (S^+^) in observing response tasks acquires secondary reinforcing properties because it is paired with food and becomes a conditioned reinforcer that then supports the acquisition of the response (i.e., S^+^ acquires appetitive, or excitatory properties). By definition, a reinforcer is an event that modifies the frequency of a response when the event is contingent on that response (e.g., Gallistel and Gibbon 2000). For example, the presentation of food is a positive primary reinforcer because when it is contingent on a lever being pressed, animals will press the lever more frequently than when the lever pressing is not paired with food. Conditioned reinforcers are initially neutral stimuli (e.g., a clicker sound) that themselves become reinforcing after having been paired with a primary reinforcer (see Mackintosh 1974, and for applications in machine learning see Sutton and Barto 2018). Thus, it has been argued that it is S^+^, once it has been associated with food, that reinforces behaviour in the observing response task and other tasks derived from it. The difficulty with this hypothesis is that by the same reasoning, the signal that is paired with a negative outcome, or ‘bad news’ (S^−^) might be expected to become a secondary conditioner for outcome absence and acquire the power to reduce the frequency of responding (i.e., S^−^ acquires inhibitory properties). If these two effects were of the same magnitude, then the preference for informative signals would not be acquired. However, if their efficiencies are of different absolute magnitude, specifically, if S^+^ increases response frequency more than S^−^ decreases it (which follows from the theoretical assumption that outcomes are more effective for learning than their absence, Rescorla and Wagner 1972; see also Murphy et al. 2001), conditioned reinforcement offers a descriptive account of the acquisition of observing responses that is not dependent on the animal being sensitive to uncertainty or its reduction.

These two hypothetical mechanisms are not, however, mutually exclusive. One postulates that information is reinforcing per se, and the other that signals for food can acquire secondary reinforcing properties because of their food contingency: both mechanisms are indeed biologically plausible. The issue is whether apparently paradoxical effects are better explained due to one factor or the other.

To arbitrate between both hypotheses, researchers have carried out cue manipulation experiments in which either the good (e.g., food) or bad (e.g., no food) outcome is no longer preceded by a signal, or in other words the presentation of either S^+^ or S^−^ is omitted (Dinsmoor 1983; Dinsmoor et al. 1972; Silberberg and Fantino 2010). According to the information hypothesis, observing responses, or preference for an informative option, should be acquired and maintained by either S^+^ or S^−^ since both resolve the uncertainty, but if conditioned reinforcement is the fundamental mechanism, they should only be acquired and/or maintained if and when the signal for good news (S^+^) is present. Though the information hypothesis is simple, functionally appealing, and intuitive, it fell broadly out of favour when evidence in pigeons began to emerge that was interpreted to be incongruent with it, but consistent with the conditioned reinforcement account. These cue manipulation experiments found that S^−^ alone was not sufficient to maintain observing responses (e.g., Dinsmoor et al. 1972; Jenkins and Boakes 1973; Kendall 1973; Silberberg and Fantino 2010). Furthermore, Roper and Zentall (1999) failed to corroborate the information hypothesis prediction that preference for the discriminative stimuli should be maximal when the degree of uncertainty is maximal (i.e., when positive and negative outcomes are equiprobable). These results, therefore, lead to the broad interpretation that information gain is not sufficient to explain observed preferences (Dinsmoor 1983; Shahan and Cunningham 2015).

Recently, however, results from protocols similar in rationale to, and derived from, the observing response protocol have rekindled interest in the possibility that animals find information intrinsically rewarding. Experiments in monkeys have found that they prefer to receive unambiguous signals about the magnitude of upcoming water rewards, over ambiguous or delayed signals, and are willing to forfeit water rewards to do so. These preferences are correlated with activity in neurons implicated in the representation of primary rewards (Blanchard et al. 2015; Bromberg-Martin and Hikosaka 2009, 2011), suggesting an intrinsic valuation of information. Furthermore, Daddaoua et al. (2016) showed that monkeys learn to actively search for Pavlovian cues to obtain conditioned reinforcement and also reduce uncertainty, though it is not yet clear how generalisable this result is to other species.

In addition, ‘paradoxical’ (also called ‘suboptimal’) choice experiments have found that pigeons (e.g., Fortes et al. 2016; González et al. 2020; Macías et al. 2021; McDevitt et al. 2018; Smith et al. 2016 also see McDevitt et al. 2016 and Zentall 2016 for reviews), starlings (Vasconcelos et al. 2015) and rats (Cunningham and Shahan 2019; Ojeda et al. 2018), prefer an alternative that provides information that they cannot use, not just when the information is neutral with respect to reward maximisation, but even when the informative option provides less reward. In this paradigm, both alternatives result in probabilistic food delivery after a delay. In the informative option, signals (S^+^ or S^−^) anticipate the trial’s forthcoming outcome immediately after a choice response, while in the non-informative option subjects are uncertain about outcomes throughout the delay. Remarkably, pigeons and starlings choose the informative option when it gives 80% less reward than the non-informative alternative (Fortes et al. 2016; Vasconcelos et al. 2015), while rats can sacrifice at least 20% of potential rewards (Cunningham and Shahan 2019; Ojeda et al. 2018) by selecting the informative option. The fact that experimental animals forfeit such significant amounts of food reward to generate apparently functionless, predictive signals is a strong reason to suspect a hypothetical primary reinforcing value of uncertainty reduction. Taken together therefore, currently available results show that the old conundrum of whether a reduction in uncertainty can by itself reinforce behaviour is still unresolved.

To investigate whether uncertainty reduction or conditioned reinforcement may better account for information-seeking behaviour we conducted paradoxical choice experiments on rats (*Rattus norvegicus)*, manipulating the salience of reward predictive cues across three treatments. All subjects were exposed to repeated choices between two options of equal average profitability. Each option delivered reward with 50% probability, a fixed time after being chosen. In all treatments, in the informative option (*Info*), the outcome of trials (food/no food) was signalled (or otherwise predictable) between each choice and the outcome, while in the other option (*NoInfo)* the outcome remained uncertain until it was realised (however, the signalling details differed between treatments, as explained below). Because the signalling occurred post-choice in *Info*, it could not be used to modify the probability of receiving food. To put it in conditioning language, the actions of choosing *Info* and *NoInfo* were both always followed by a 50% probability of a food outcome.

In the *S*^+^*_S*^*−*^ treatment, the interval between choosing *Info* and the outcome was filled in rewarded or unrewarded trials by either of two sounds, namely S^+^, or S^−^ cues, respectively. In the *Only_S*^*−*^ treatment, the interval was silent in trials when food was due but filled with a sound when no food was coming (i.e., there was no explicit S^+^ cue). In the *Only_S*^+^ treatment, the same interval was filled with a sound signal in trials when food was forthcoming and with silence when it was not (i.e., there was no explicit S^−^ cue).

The information hypothesis predicts that both S^+^ and S^−^ individually, should be sufficient to generate observing responses, because both provide information about an otherwise uncertain outcome. The conditioned reinforcement hypothesis, on the other hand, stipulates that only S^+^ should be positively reinforcing. According to this view, although S^−^ reduces uncertainty just as much as S^+^, its presence should reduce rather than increase the acquisition of an observing response or preference for an informative option.

Strong support for the conditioned reinforcement hypothesis would be corroborated if a preference for the informative option were recorded when the signal for no reward is omitted (*Only_S*^+^ treatment) but not when the signal for sure reward is omitted (*Only_S*^*−*^ treatment), as this would show that a salient S^+^ is both necessary and sufficient for the development of *Info* preference. On the other hand, if subjects developed an equally strong preference for the informative option regardless of whether S^+^ or S^−^ were omitted, the results would be consistent with the predictions of the information hypothesis, because both resolve uncertainty to the same degree regardless of their valence.

Manipulations of the signalling properties of choice alternatives in the paradoxical choice procedure have been performed previously in pigeons and starlings (e.g., Fortes et al. 2017; Vasconcelos et al. 2015 and see McDevitt et al. 1997 for similar tasks), but we found no reports of the relative quantitative impact of symmetrical omissions of S^+^ and S^−^, the most distinctive prediction of the two hypotheses. Thus, our experiment offers novel insights into the putative mechanisms underlying information-seeking behaviour.

We recorded two measures of preference, namely proportion of choices in 2-option choice trials, and response latencies in 1-option forced trials. Response latencies have proven to be a robust metric of preference in a variety of different behavioural protocols and species (viz. Kacelnik et al. 2011; Monteiro et al. 2020; Reboreda and Kacelnik 1991; Sasaki et al. 2018; Shapiro et al. 2008; Smith et al. 2018).

## Methods

### Subjects

Twenty-four male Lister Hooded rats (*Rattus norvegicus,* provider Envigo), 11 weeks old at the start of the experiment served as subjects. We used an all-male cohort to reduce inter-individual variability and temporal within-subject variability, but it is worth noticing that it is possible that exploratory (information-seeking) behaviour in females varies adaptively through the estrous cycle, and this is an important topic in itself. Animals were housed in groups of four. Throughout the experiment, subjects were food deprived to a minimum of 85–90% of their expected free-feeding weight using growth curves from the provider. Initial weight: 337 ± 14, final weight: 357 ± 16 (mean ± std.) Water was provided ad libitum in their home cages, and they were maintained on a 12-h dark/light cycle with lights on at 6 AM.

### Apparatus

Testing was carried out in eight operant chambers (Med Associates, USA.) Each chamber contained three retractable levers: one in the back panel (centre) and two in the front panel, left and right of a central food magazine. The magazine was equipped with an infrared beam and a sensor to record head entry. Each reward delivery consisted of four 45 mg sucrose pellets (TestDiet, USA). A speaker was positioned above the food magazine in the front panel. Each chamber was also equipped with a houselight (white) and a fan, which were switched on for the duration of the session. The chambers were controlled via custom-written Med-State Notation programs running on MED-PC V (Med Associates, USA).

### Training

#### Magazine training

To habituate the rats to the box and the delivery of food rewards, training began with a single variable interval session where food was delivered on average once a minute (VI60 free food schedule) a total of 60 times. The variable interval was sampled from a truncated Poisson distribution with a mean of 60 s and range of 0–120 s.

#### Lever training

Over the next three sessions, the rats were trained to press the two front levers. Either lever (left or right with equal probability) was available on each trial (60 trials per session). Once a lever extended into the chamber, a single press resulted in its retraction and immediate reward delivery (Fixed Ratio 1 schedule). One of the levers then again became available after a delay composed of a constant duration plus a variable one. The constant component was 5 s, and the variable one was sampled from a truncated Poisson distribution with a mean of 20 s and a range of 5–60 s. All three sessions concluded after 60 reward deliveries, i.e., 30 lever presses on each side, or after 3 h.

#### Cues

Within the main experiment and training there were four auditory cues, all with a duration of 10 s, and each associated to a reward probability. There were two cues for the informative option: S^+^ (100% reward probability) and S^−^ (0% reward probability), and two cues for the non-informative option: N1 and N2 (both with 50% reward probability). The four sounds were: a low-frequency pure tone (3 kHz, 78 dB), a high-frequency pure tone (6 kHz, 78 dB), a buzzing sound (78 dB) and a clicking sound (74 dB). Assignment of sounds to reward probabilities was counterbalanced across subjects to avoid the possibility of option preferences being influenced by any intrinsic aversive or attractive properties of the sounds.

#### Cue training

To train the subjects to the reward contingencies of the four auditory cues (S^+^, S^−^, N1, and N2), the main experiment was preceded by a Pavlovian protocol in which all the rats were exposed to the cues and their respective reward contingencies. In this phase, cue presentation was independent of the behaviour of the rat. These cue-training sessions consisted of 40 trials, with 10 trials for each of the 4 cues, intermixed in random order. To avoid large deviations from the expected outcome probabilities of cues N1 and N2 in each session, proportions of outcomes were fixed as one half for each cue. Trials were separated by an ITI generated by sampling from of a truncated Poisson distribution with a mean of 50 s (range 10–120 s) + 10 s (to ensure a minimum ITI of 20 s; range: 20 s ≤ ITI ≤ 130 s). Subjects performed one daily session of this phase for 10 days. Cumulative time spent head-poking into the food magazine was measured to establish the degree of cue discrimination.

### Experimental procedures

#### General procedure

**Fig. 1:**
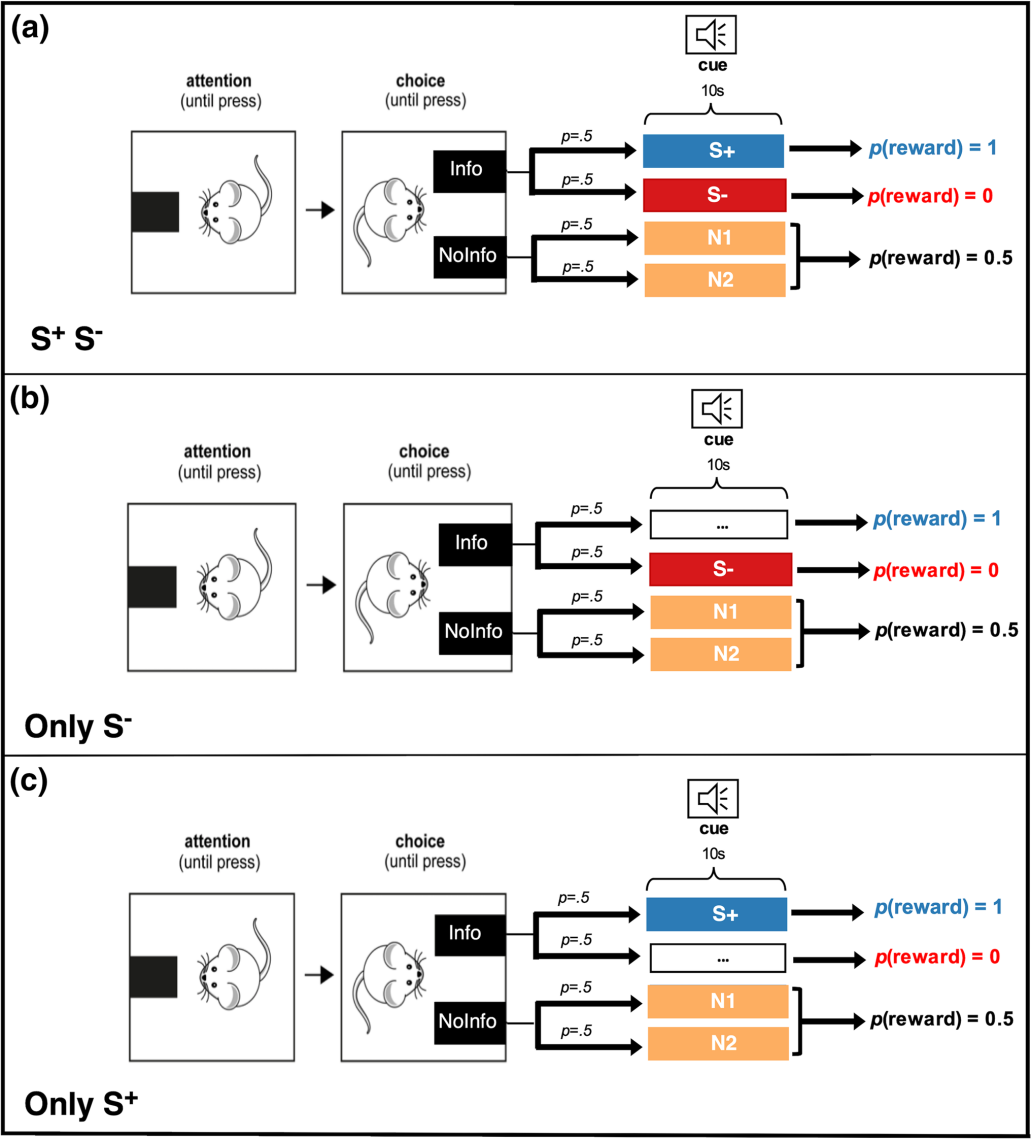
Experimental design showing choice trial structure for each treatment. Blank boxes with ellipsis indicate no auditory signal (silence) preceding outcomes. *p* denotes probability

We used a trial-based chain procedure as displayed in Fig. 1. There were two kinds of trials: 2-option choice trials and 1-option forced trials. A day’s session was composed of 60 trials: 40 forced (half *Info* and half *NoInfo*) and 20 choice, which were randomly intermixed. All trials started with the rear lever extending. Pressing this lever resulted in its retraction, and either one (forced trials) or both (choice trials) of the front levers being presented. Pressing a front lever could initiate an acoustic cue and the retraction of that lever (forced trials) or of both levers (choice trials). The auditory cue, if present, was broadcast for a 10 s interval, after which food delivery occurred in rewarded trials without the need for a further response. Thus, each option was programmed as a discrete trial, response initiated, fixed time 10 s, partial reinforcement 50% schedule. Trials were separated by an inter-trial interval (ITI) generated by sampling from a truncated Poisson distribution with a mean of 50 s (range: 10–120 s) and adding 10 s. A session finished after 60 trials or 3 h, whichever occurred first.

#### Experimental procedure

In the *S*^+^*_S*^*−*^ treatment, choosing *Info* resulted with equal probability in either S^+^, which was paired with reward, or S^−^, which was paired with no reward, thereby reliably informing the subject of the forthcoming outcome. Pressing the *NoInfo* lever, on the other hand, resulted with equal probability in either of two cues: N1 or N2, which were both associated with a 50% probability of either outcome; therefore, neither cue informed the subject of forthcoming reward.

The other two treatments, *Only_S*^+^ and *Only_S*^*−*^, differed from the *S*^+^*_S*^*−*^ treatment only in the signalling properties of *Info*. In *Only_S*^*−*^ responding to *Info*, resulted with equal probability (50%) in either a 10 s silence, followed by the delayed reward (the omission of a cue associated with reward, i.e., S^+^), or the auditory S^−^ cue, which was associated with no reward. In *Only_S*^+^ choosing *Info* resulted with equal probability (50%) in either the cue *S*^+^*,* which was associated with reward after 10 s, or a 10 s silence (omission of the S^−^ cue) followed by no reward (Fig. 1).

A between-subject design was used, with eight rats in each group. Subject assignment to group was organised such that there was no correlation between group and any of the following parameters: side of the informative option; hour of testing; cage in which the animals were housed, or cue–reward contingencies. For each group, the subjects performed one daily session for 14 days. Each rat was trained at the same time every day; one cohort of rats began the experiment at 9:00 AM, another at 12:30 PM, and the last at 3:30 PM.

## Data analysis

Data processing and analysis was carried out in MATLAB 2017a and statistical tests were carried out with R statistical software (https://www.r-project.org; Version 1.2.5033). A type-1 error rate of 0.05 was adopted for all statistical comparisons and the Tukey test was used for all multiple comparisons. For statistical analysis, choice proportion data were arc-sine square-root transformed to normalize the residuals. Head-poking data as well as latency index data, were square root transformed (Grafen and Hails 2002). For all analyses of head-poking data, data from both stimuli signalling 50% chance of reward were averaged for each subject.

Mean choice proportion data for each treatment group were fitted with sigmoidal curves using the following function:

1 $$\Psi \left( {x; \; \alpha ,\beta ,\gamma ,\lambda } \right) = \gamma + \left( {1 - \gamma - \lambda } \right)F_{{{\text{Gauss}}}} \left( {x; \; \alpha ,\beta } \right),$$

where ${F}_{\mathrm{Gauss}}\left(x;\alpha ,\beta \right)$ is a cumulative Gaussian function. Non-linear least squares was used and implemented with the FitPsycheCurveWH function in MATLAB (Wichmann and Hill 2001). λ and $\gamma$ set the upper and lower bounds of the curves respectively while $\alpha$ gives the inflection point and $\beta$ the slope at this value of *x*. The upper bound was set at 1 for all curves while other parameters were estimated (Table S1).

To measure preference on the basis of latency to respond in forced trials, for each individual we calculated an index, *L*_*(Info)*_*,* using the median latencies to respond on *Info* (*R*_*(Info)*_) and *NoInfo* (*R*_*(NoInfo)*_) forced trials for each session: *L*_*(Info)*_ = *R*_*(Info)*_ / (*R*_*(Info)*_ + *R*_*(NoInfo)*_). Values of *L*_*(Info)*_ < 0.5 or *L*_*(Info)*_ > 0.5 indicate a preference for *Info* or *NoInfo* respectively, as measured in forced trials, independently of the measure of preference based on choices in 2-option trials.

### Ethical note

All experiments were carried out in compliance with the UK Animal (Scientific Procedures) Act (1986) and its associated guidelines.

## Results

### Training

#### Cue discrimination

**Fig. 2:**
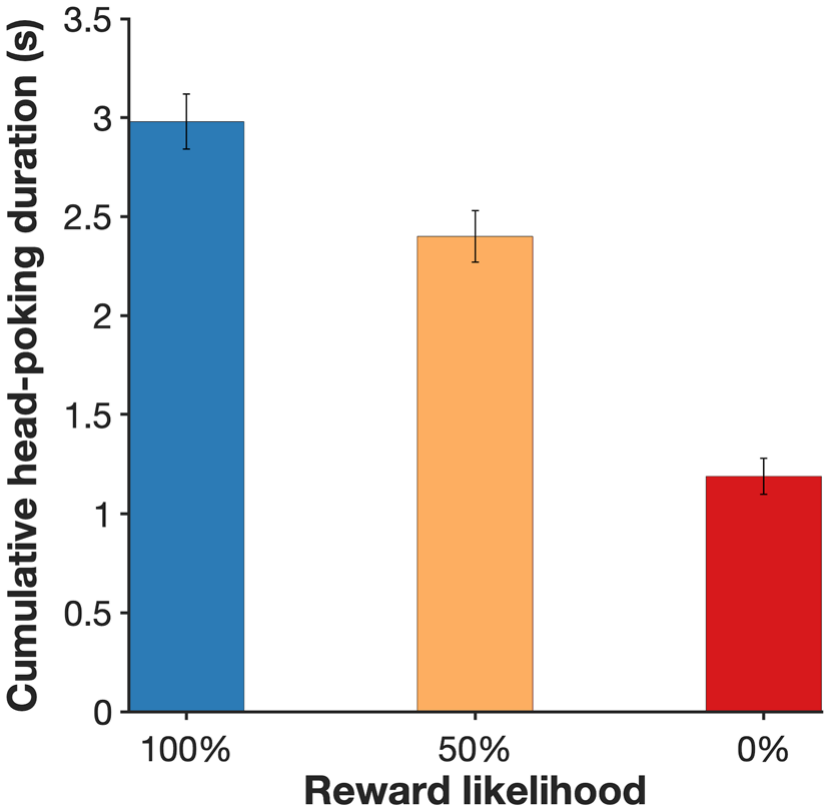
Time spent head-poking into the food magazine during cue presentation at the end of the training phase. The data shows the mean cumulative time (mean ± s.e.m.) subjects spent with their head in the food magazine over the 10 s intervals preceding reward outcomes, pooled from the last three sessions of training. During this time reward-predictive signals indicating a 100%, 50% or 0% chance of reward were presented (corresponding to S^+^, N1 or N2, and S^−^, respectively). *n* = 24

A condition for the interpretation of preferences is that subjects were able to discriminate the contingencies of each cue; we examined this using cumulative head-poking time during the 10 s interval between choice and outcome, when the cues were present, pooling data from the last three training sessions across the groups, which up to that point had no differential experience (Fig. 2). The cue associated with 100% reward probability (S^+^) had the longest cumulative head poking duration (2.98 s ± 0.14; mean ± s.e.m.), followed by the mean of both cues associated with 50% probability (N1 & N2: 2.40 s ± 0.13), while the cue associated with no reward (S^−^) elicited the shortest average head poking duration (1.19 s ± 0.09). A one-way repeated-measures ANOVA revealed a significant effect of cue (F_2,46_ = 44.38, *P* < 0.0001). Post hoc pair-wise comparisons showed a significant difference between all pairs (100% vs 50%, *P* < 0.05; 100% vs 0%, *P* < 0.001 and 50% vs 0%, *P* < 0.001). This confirms that subjects discriminated the contingencies programmed for each cue.

### Experiment

#### Preference 1: Choice in 2-option trials

**Fig. 3:**
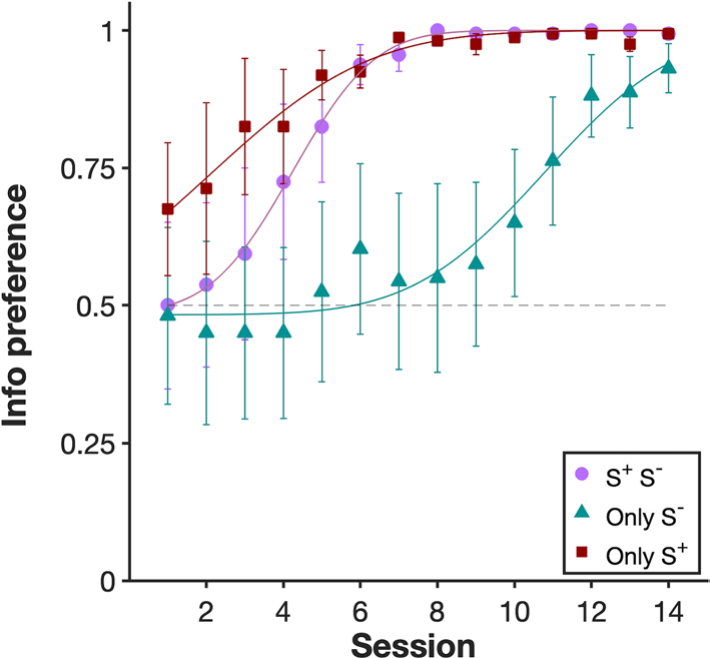
Preference for the *Info* option in choice (2-option) trials. Proportion of observed choices for the *S*^+^*_S*^*−*^ (*n* = 8), *Only_S*^*−*^ (*n* = 8) and *Only_ S*^+^ (*n* = 8) groups are shown (means ± s.e.m.) Lines are sigmoidal curves with a cumulative Gaussian fit (see methods for details). See supplementary materials (Table S1) for parameter estimates of each fitted curve

In choice trials, a strong preference for *Info* developed in all three treatments, with acquisition occurring more slowly in the *Only_S*^*−*^ treatment (Fig. 3, Fig. S1). A two-way repeated-measures ANOVA on data across all sessions with treatment as a between-subject factor, session as a within-subject factor, and (transformed) proportion of choices for *Info* as the response variable, revealed significant effects of treatment (F_2,21_ = 4.00, *P* < 0.05) and session (F_13,276_ = 14.4, *P* < 0.0001), and a significant interaction (F_26,273_ = 1.56, *P* < 0.05), reflecting the slower acquisition in the *Only_S*^*−*^ treatment. Given the significant interaction, and the plot in Fig. 3, it is obvious that the main effects are caused by rate of acquisition and not by asymptotic levels.

To establish whether the acquired preferences for *Info* were stable rather than transient, we analysed preferences at the end of the experiment by pooling data over the last three sessions. In all three treatments, the animals showed a strong preference for the informative option for as long as they were tested: 99.8% ± 0.002 (mean ± s.e.m.) in the *S*^+^*_S*^*−*^ treatment*;* 90% ± 0.030 in *Only_S*^*−*^*,* and 98.8% ± 0.005 in *Only_ S*^+^*.* These values are all significantly greater than 50% (t_7_ = 47.5, *P* < 0.0001; t_7_ = 5.86, *P* < 0.001; and t_7_ = 22.8, *P* < 0.0001, respectively).

#### Preference 2: latency in 1-option trials

**Fig. 4:**
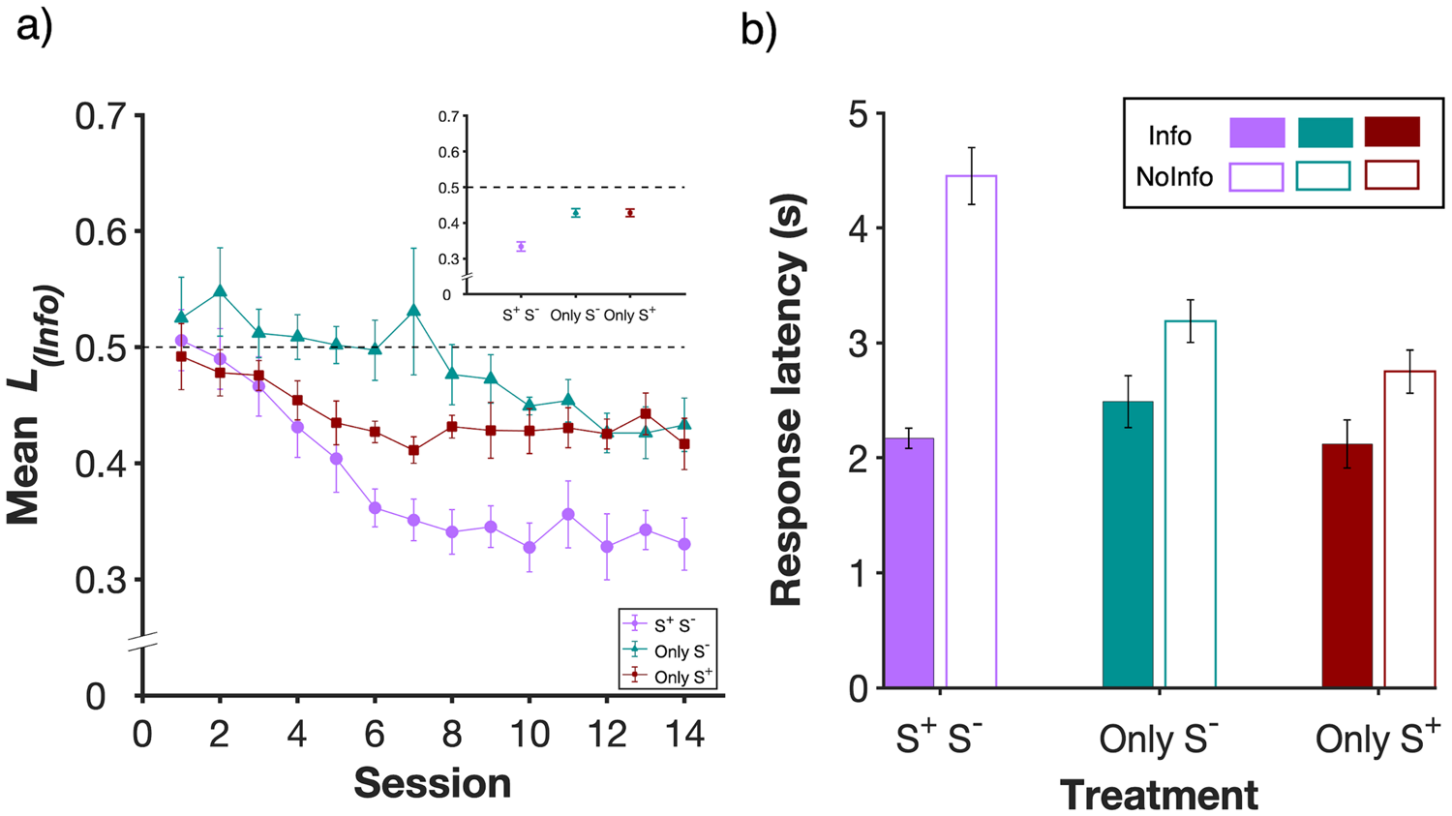
Latency to respond to *Info* vs *NoInfo* in forced (1-option) trials. **a** Latency-based preference index for all three treatments where *L*_*(info)*_ = *R*_*(Info)*_ / (*R*_*(Info)*_ + *R*_*(NoInfo)*_), and *R*_*(Info)*_ and *R*_*(NoInfo)*_ are the median latencies to respond in *Info* and *NoInfo,* respectively. *L*_*(Info)*_ values below 0.5 indicate preference for *Info* while values of *L*_*(Info)*_ above 0.5 indicate preference for *NoInfo.* The inset shows data pooled over the last 3 sessions. *n* = 8 in each group. **b** Filled bars show latency to respond to *Info* (*R*_*(Info)*_) and unfilled bars show latency to respond to *NoInfo* (*R*_*(NoInfo)*_) across the three treatments (means ± s.e.m.), with data pooled from the last three sessions. *n* = 8 in each group

In the previous section, we measure preference using proportion of choices in trials when both alternatives were present, here we use latency to respond in single-option forced trials. This is the time between a subject initiating a trial by pressing the back lever and pressing the lever for the single option that subsequently becomes available. Latencies or reaction times in 1-option trials have proven to be a robust predictor of choice in 2-option trials, and are very informative with respect to the psychological mechanism of choice (see, for instance, Monteiro et al., 2020). Since in each session each individual completed 20 *Info* and 20 *NoInfo* forced trials, we used the median latency shown by each individual for each alternative for analysis. Figure 4a shows that latencies in single-option trials mirrored the rats’ choice proportions in choice trials: in all treatments, latencies were shorter in *Info* than *NoInfo* in the final sessions of the experiment. The absolute value of latencies is shown in Fig. 4b and reveals that while latency to respond to *Info* was fairly constant across treatments, latency towards *NoInfo* varied: it was very long in the *S*^+^*_S*^*−*^ treatment, intermediate in *Only_S*^*−*^ and shortest in *Only_S*^+^. This is interesting because *NoInfo* was identically programmed across treatments; we return to this point in the discussion.

To quantify the acquisition of preference using forced trials data, we ran a two-way repeated-measures ANOVA on data across all sessions, with treatment as a between-subject factor, session as a within-subject factor and latency-based preference index *L*_*(Info)*_ as the dependent variable (see Methods). This revealed a significant effect of treatment (F_2,21_ = 9.72, *P* < 0.01), session (F_13,273_ = 17.54, *P* < 0.0001), and a significant interaction (F_26,273_ = 2.97, *P* < 0.0001).

Post hoc pair-wise comparisons on data pooled from the last 3 sessions showed that while *L*_*(Info)*_in *Only_S*^*−*^ (0.43 ± 0.01 mean ± s.e.m.) and *Only_S*^+^ (0.43 ± 0.01) were not significantly different from each other (*P* = *1*), *L*_*(Info)*_in both of these groups was significantly higher compared to the *S*^+^*_S*^*−*^ group (0.33 ± 0.01; *P* < 0.0001 in both cases). Further, consistently with a preference for *Info*, over the last 3 sessions *L*_*(Info)*_was significantly lower than 50% in all treatments (*S*^+^*_S*^*−*^: t_7_ = − 7.02, *P* < 0.001; *Only_S*^*−*^*:* t_7_ = − 4.74 *P* < 0.01; *Only_S*^+^: t_7_ =  − 4.16, *P* < 0.01), indicating that also on this metric subjects preferred *Info*. In other words, according to this index, preference for *Info* persisted at the end of training, and was strongest when both outcomes were explicitly signalled, and equally strong when either of the outcome signals was omitted.

#### Head-poking during cue presentation

**Fig. 5:**
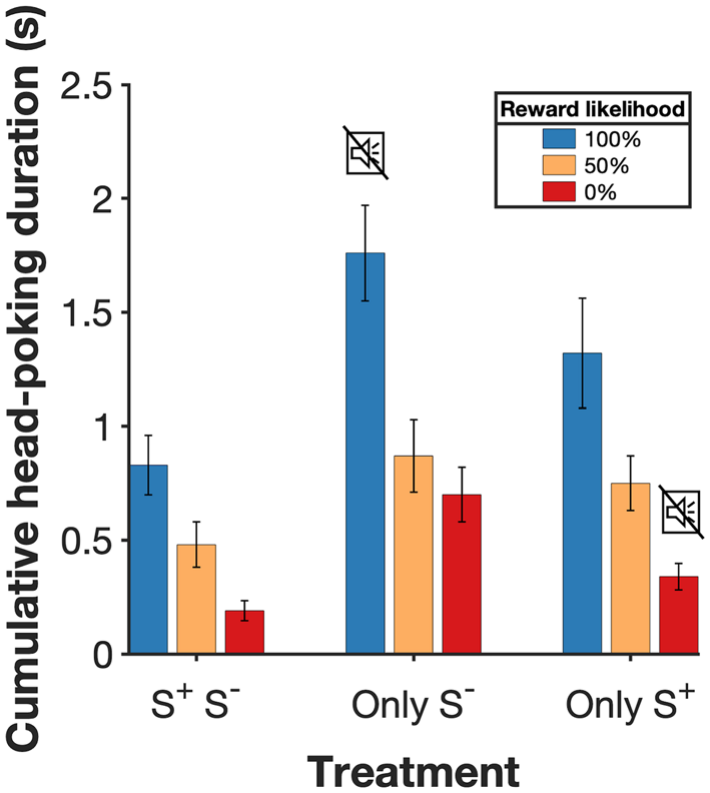
Time spent head-poking into the food magazine between choice and outcome in the main experiment. The bars show the average cumulative time subjects spent with their head in the food magazine in the 10 s preceding reward outcomes (± s.e.m.), pooled over the last 3 sessions. During this time reward-predictive signals indicating a 100%, 50% or 0% chance of reward could be presented. Data for the *S*^+^*_S*^*−*^ (*n* = 8), *Only_S*^*−*^ (*n* = 8) and *Only_S*^+^ (*n* = 8) groups are shown. The muted speaker symbol indicates that an explicit cue was not used to signal a particular outcome

Although during the main experiment behaviour post-choice did not influence outcomes, rats anticipated food by head-poking into the food magazine (possibly a Pavlovian response). Data from choice and forced trials show that in the *Info* option, subjects head-poked more in trials when food delivery was due than when it was not and showed an intermediate level of head-poking in *NoInfo*, when there was a 50% chance of food delivery (Fig. 5).

Pooled over the last three sessions, time spent head-poking into the food magazine ranked as reward probability (100% > 50% > 0%). This was the case in all treatments: the *S*^+^*_S*^*−*^ group (100%: 0.83 s ± 0.13; mean ± s.e.m., 50%: 0.48 s ± 0.1, 0%: 0.19 s ± 0.04), *Only_S*^*−*^ (100%: 1.76 s ± 0.21, 50%: 0.87 s ± 0.16, 0%: 0.70 s ± 0.12), and *Only_S*^+^ (100%: 1.32 s ± 0.24, 50%: 0.75 s ± 0.12, 0%: 0.34 s ± 0.06).

A two-way ANOVA on these data with reward probability as a within-subject factor, treatment as a between-subject factor and cumulative head-poking as the response variable revealed a significant effect of reward probability (100%, 50% or 0% reward; F_2,42_ = 27.80, *P* < 0.0001) but not treatment (F_2,21_ = 2.53, *P* = 0.104) and no significant interaction (F_4,42_ = 0.51, *P* = 0.732). Post hoc pair-wise comparisons showed that head-poking was significantly higher when reward was due (100%) than when it was not (0%) in all treatments (highest *P* < 0.001). The fact that head-poking reflected forthcoming reward outcomes differentially in S^+^ and S^−^ trials regardless of treatment shows that rats recognized the current contingency regardless of whether an explicit cue was present. The absolute level of head-poking seemed to be inversely related to how much food signalling was available (*Only_S*^*−*^ > *Only_S*^+^  > *S*^+^*_S*^*−*^), as if attention to explicit signalling competed with exploratory investigation of the food magazine, though this is a non-significant trend.

## Discussion

We explored the role that two putative psychological mechanisms—uncertainty reduction and conditioned reinforcement—have in determining preference for an informative option in which delayed outcomes are signalled by predictive cues, over an equally profitable non-informative option, in which outcomes remain uncertain until they are realised. A pre-existing observation that is considered to be functionally paradoxical and mechanistically unclear, is that in such protocols animals show a strong bias for the informative option, even though the information they gain is non-instrumental, i.e., cannot be used to modify outcomes and increase rewards. As an aside, note that we label this preference as being paradoxical (which relates to the observer’s expectations) and not, as it is frequently done, ‘suboptimal’ (see Zentall 2016) which implies that the mechanism generating the behaviour is maladaptive in ecological contexts, a misleading and unsupported interpretation.

We relied on two independent metrics of preference: proportion of choices in 2-option trials, and response latency in 1-option trials. As we show below, this helps to judge the robustness of preferences and to unravel behavioural mechanisms. In the *S*^+^*_S*^*−*^ treatment, where we reproduced the classic ‘paradoxical choice’ protocol. Our results are consistent with previous studies in rats: when presented with two options that differ only in the post-choice predictability of delayed outcomes, rats (as birds and primates) strongly prefer the more informative alternative (Chow et al. 2017; Cunningham and Shahan 2019; Ojeda et al. 2018). This was observed both in proportion of choices between the alternatives and in differential response latencies when only one of them was present. Some previous studies on rats failed to find preference for the informative option (Alba et al. 2018; López et al. 2018; Martínez et al. 2017; Trujano et al. 2016; Trujano and Orduña 2015), though this is likely because in these studies *Info* had a lower probability of reward than *NoInfo*, whereas in our study both options were equally profitable, and unlike pigeons and starlings, rats are very sensitive to reward losses incurred by selecting *Info* (Fortes et al. 2016; Ojeda et al. 2018; Smith et al. 2016; Vasconcelos et al. 2015).

Our results show that asymptotic *Info* preference is robust to the absence of an explicit, salient good news (*Only_S*^*−*^ treatment), or bad news (*Only_S*^+^ treatment) stimulus (Fig. 3). When the period preceding reward or no reward, respectively, was filled with silence rather than an auditory cue, subjects still developed a strong preference for *Info* in both cases. Our finding that a salient S^+^ is not necessary for *Info* preference is consistent with similar observations in starlings (Vasconcelos et al. 2015), monkeys (Lieberman 1972) and humans (Fantino and Silberberg 2010; Lieberman et al. 1997). Figure 3 does show, however, that the absence of a salient S^+^ in *Only_S*^*−*^ slows the acquisition of *Info* preference relative to the standard *S*^+^*_S*^*−*^ treatment, while the absence of a salient S^−^ in *Only_S*^+^ has a much weaker, although positive, effect on the speed of acquisition. This result is congruous with several studies indicating that S^+^ has a more significant impact on preference in paradoxical choice than S^−^ (Fortes et al. 2017; Laude et al. 2014; McDevitt et al. 1997; Pisklak et al. 2015; Spetch et al. 1994) and also those showing that in rats S^−^ acquires inhibitory properties (Alba et al. 2018; Martínez et al. 2017; Trujano et al. 2016; see González and Blaisdell 2021 for evidence of this in pigeons).

Our analysis of preference on the basis of latency in single option trials is inspired by the Sequential Choice Model (SCM; Kacelnik et al. 2011; Monteiro et al. 2020; Shapiro et al. 2008). The SCM postulates that choice can be modelled as a horserace between the latency distributions of available alternatives because the alternatives are psychologically processed in parallel, without an active process of choice. Measuring behaviour by more than one procedure is in itself important, because if the phenomenon being measured is meaningful, it should show procedural invariance, a property often claimed to be violated by studies of human preferences using choice Vs. willingness to pay (Slovic 1995). We did find consistency between our measures of preference, but also found that using response latency as an additional metric informed about important aspects of potential underlying mechanisms. As Fig. 4 shows, response latencies in forced trials for *Info* were consistently shorter than in trials for *NoInfo*, across treatments. Variations between treatments were mediated only by latency differences in *NoInfo*, which was identically programmed in all three treatments. In other words, treatment effects were mediated by modifications of latency to respond to the least preferred alternative. This result is striking, could not have been anticipated by the choice results, and is consistent with what was reported by Smith et al. (2018) in a midsession reversal protocol with pigeons, a very different experiment, and species. They too found that changes in choice proportions were explained by variations in latency towards the least preferred alternative in single option trials, when that option did not itself change in its properties. It seems appropriate to infer that parallel processing of alternatives, and mediation through latency variation in less preferred alternatives can be widespread properties of choice behaviour, something that the analysis of choice, which is prevailing in studies of preference, could not have revealed.

We focused on two potential psychological hypotheses about the mechanisms supporting the observed bias for the informative option. The information hypothesis contends that individuals treat uncertainty as aversive, so that informative signals, regardless of whether they bring good or bad news, drive preference acquisition. In contrast, the conditioned reinforcement hypothesis argues that preference for the *Info* option increases due to signals for food (S^+^, ‘good news’) and decreases due to signals for food’s absence (S^−^, ‘bad news’), because S^+^ acquires secondary excitatory properties, and S^−^ inhibitory properties, with the excitatory influence of S^+^ deemed to be greater than the inhibitory effect of S^−^.

Though both mechanisms are plausible, both run into functional difficulties. For the information hypothesis, this is that in the experimental situation, acquiring information does not give subjects the ability to increase reward outcomes (but see Dinsmoor 1983 for the suggestion that it may improve the usefulness of outcomes). This difficulty, like other experimental observations of so-called suboptimal or irrational behaviour, can be addressed post hoc by arguing that in nature, information about relevant commodities is very often likely to be usable, either immediately or further in the future, so that evolution may design utility functions that are somehow tricked by the experimental protocols. For example, foraging-inspired theoretical models (Freidin and Kacelnik 2011; Vasconcelos et al. 2015) have argued that in nature, information, even if it announces unfavourable events, is likely to be immediately useful: an animal that knows for sure that the prey being presently pursued will not be captured, would abort the chase, and thus would not pay the opportunity cost of waiting for a null outcome. Thus, Vasconcelos et al. (2015) have argued that in the lab animals do not include the delays associated with S^−^ in their profitability computations, because they would normally use such time beneficially. Furthermore, even if the information cannot be used straight away, acquiring it may help to solve novel problems in the future (Gottlieb and Oudeyer 2018). In other words, it is the artificiality of being unable to use information in the experimental protocol that generates the paradox, which can be reconciled by considering the ecological context in which the mechanism of behaviour evolved (Vasconcelos et al. 2018). Similarly, with the conditioned reinforcement hypothesis, while there are no a priori reasons why the excitatory effect of S^+^ should be greater than the inhibitory effect of S^−^, it is likely that in nature cues indicating the presence of relevant commodities are more prevalent or reliable than those indicating their absence. Therefore, the power of excitatory and inhibitory conditioned stimuli to modify behaviour need not be symmetric (e.g., Rescorla and Wagner 1972). Placing the phenomenon in its natural ecological circumstances may be key to promote its understanding.

Our results do not lend unequivocal, exclusive support to either the conditioned reinforcement hypothesis or the information hypothesis in their original formulations. However, an extended conditioned reinforcement account (which we detail below), or both mechanisms acting simultaneously, may explain our results. We discuss these putative explanations below, focussing first on the information hypothesis.

The information hypothesis makes two predictions that distinguish it from the conditioned reinforcement account. The first is that S^−^ on its own should reinforce *Info* preference. Our main result is consistent with this prediction: in the *Only_S*^*−*^ treatment group where a salient S^+^ was absent, but S^−^ present, rats also acquired a strong preference for *Info,* which could be interpreted to show that S^−^ reinforces *Info* responses, rather than just inhibiting them. Head-poking data from the delay period between choice and reward outcomes, however, could be taken to suggest otherwise. Across all treatments we found that time spent head-poking into the food magazine ranked as reward probability (100% > 50% > 0%; Fig. 5). In other words, when an explicit S^−^ cue was present, magazine head-poking was lower than when S^+^ or an uninformative cue were present, indicating that S^−^ may have inhibitory properties. Note though that the fact that S^−^ may inhibit post-choice head-poking does not necessarily preclude it from reinforcing *Info* choice (which occurs earlier in the trial) via uncertainty reduction.

A quantitative prediction of the information hypothesis is that S^+^ and S^−^ should reinforce choices for *Info* to the same extent. This is because both stimuli in the informative option completely resolve the pre-choice uncertainty; hence they convey the same amount of information. Thus, if uncertainty reduction were the only consideration, S^+^ and S^−^ would be equally reinforcing. Our finding that the omission of an explicit S^+^ slows acquisition to a greater extent than the omission of an explicit S^−^ is incongruent with this prediction. Taken together therefore, our results cannot be fully explained by the information hypothesis alone.

According to the conditioned reinforcement account, animals prefer the informative option because of the excess excitatory effect of good news. Explanations of precisely how S^+^ can acquire value as a conditioned reinforcer in paradoxical choice have been developed by several different authors and include: the Contrast Hypothesis (Case and Zentall 2018; Gipson et al. 2009; Zentall 2013; see also González et al. 2020 for a hypothesis that considers contrast but not conditioned reinforcement per se), the Stimulus Value Hypothesis (Smith et al., 2016; Zentall et al. 2015; Smith and Zentall 2016), the Signals for Good News (SiGN) Hypothesis (Dunn and Spetch 1990; McDevitt et al. 2016), the Temporal Information Model (Cunningham and Shahan 2018 though note that their model also considers how primary reinforcement affects choice), and the Selective Engagement Hypothesis (Beierholm and Dayan 2010; Dinsmoor 1983).

We do not have the scope or the data to examine and differentiate these hypotheses in detail, but they all share the assumptions that (1) S^+^ alone is responsible for acquisition and maintenance of *Info* preference, and (2) the excitatory effect of S^+^ is greater than the inhibitory effect of S^−^ (with some claiming that S^−^ has no effect at all). The conditioned reinforcement account on its own therefore predicts that preference for *Info* can only develop when there is a perceivable S^+^ cue in *Info* capable of acquiring reinforcing properties.

The fact that subjects in the *Only_S*^*−*^ treatment, where there was no explicit auditory S^+^ signal, developed preference for *Info*, *prima facie* contradicts the conditioned reinforcement account, as the result suggests that S^+^ is not necessary for the development of *Info* preference. However, an elaborated conditioned reinforcement account could explain our results, and we discuss this below.

It could be argued that the manipulation we performed was not sufficient to eliminate the putative positive conditioned reinforcement afforded by the informative option. After all, head poking data showed that during the post-choice delay rats could anticipate whether food was imminent, even for outcomes not signalled by a salient cue (Fig. 5). A conditioning explanation for this could be that subjects treat the compound of their action (lever pressing) plus the immediate absence of a salient cue as a predictive event or conditioned stimulus (CS) in itself, and the delayed outcome (food or no food) as the unconditioned stimulus (US). Thus, they could learn the pairing *[Press Info* + *Silence]* → *food* in the *Only_S*^*−*^ treatment, while those in *Only_S*^+^ could learn *[Press Info* + *Silence]* → *no food*. A Pavlovian version of the same idea is that the CS compound does not comprise just the rat’s action, but also the lever retraction that follows from it. It is therefore possible that in the *Only_S*^*−*^ group, *Info* lever pressing/retraction followed by the *absence* of an auditory cue is a compound stimulus used by rats to anticipate reward, in other words, it is a virtual S^+^. Under this rationale, conditioned reinforcement can be present in *Info* even with no salient perceptual cue precedes rewards, and thus could account for the development of preference for *Info* in our experiment.

Paired with another well-established psychological phenomenon, the feature-positive effect (Crowell and Bernhardt 1979; Newman et al. 1980; Pace et al. 1980; Sainsbury 1971), this elaboration of the conditioned reinforcement hypothesis could provide a full explanation of both preference and speed of acquisition across our treatments. The feature-positive effect refers to the observation that in discrimination learning involving the presence or absence of a feature, subjects find it easier to associate the presence of a feature with a positive outcome (S^+^) than they do the absence of the same feature. This could explain why subjects developed *Info* preference faster in *Only_S*^+^ compared with *Only_S*^*−*^. In *Only_S*^*−*^ food rewards in *Info* are preceded by the lack of an auditory cue; a feature negative stimulus, while in *Only_S*^+^ food is preceded by an explicit, feature-positive, auditory cue. Therefore, the feature negative silence in *Only_S*^*−*^ would have taken longer to acquire secondary reinforcing properties than the feature positive reward cue in *Only_S*^+^, which may explain the difference in acquisition between the treatments. This explanation means that we cannot rule out conditioned reinforcement as the primary underlying mechanism driving *Info* preference in our experiment.

Finally, our results are also consistent with the possibility that both uncertainty reduction and conditioned reinforcement act simultaneously to generate preference in paradoxical choice (see Daddaoua et al. 2016 for a similar argument in monkeys). An asymptotic preference for *Info* that is robust to the absence of an explicit S^+^ cue is consistent with the information hypothesis prediction that a perceivable S^−^ alone is sufficient to generate *Info* preference via uncertainty reduction. Additionally, the faster acquisition in *Only_S*^+^ where there is an explicit S^+^ compared to *Only_S*^*−*^ where there is not, supports the conditioned reinforcement hypothesis’ assumption that a perceivable S^+^ reinforces *Info* choices. Taken together intrinsic information value and conditioned reinforcement can therefore provide a reasonable account of our results and those of other studies. This mechanism would capture as significant two functionally relevant commodities—both the *amount* of information and its *content* (i.e., good news or bad news)—as factors that shape the acquisition of preferences in the paradoxical choice protocol.

In summary, we found that rats show a robust preference for advanced non-instrumental information and that this preference is more strongly influenced by good news than bad news. Counterintuitively, treatment effects were mediated by differences in the latency to respond to the least preferred alternative, which was identical across all treatments. Our results show that while uncertainty reduction is unlikely to solely account for preferences for advanced information, the balance of evidence indicates that it may play some role alongside conditioned reinforcement.

## Supplementary Information

Below is the link to the electronic supplementary material.

Supplementary file1 (PDF 320 KB)

Supplementary file2 (XLSX 29 KB)

Supplementary file3 (XLSX 17 KB)

Supplementary file4 (XLSX 20 KB)

Supplementary file5 (XLSX 38 KB)

Supplementary file6 (DOCX 182 KB)

## Data Availability

Data can be found in the supplementary materials. Code for analysis can be made available on reasonable request to the corresponding authors.
