# Supplementary material for: Paradoxical choice and the reinforcing value of information
Source: Anim Cogn. 2022 Oct 28;26(2):623–37. doi: 10.1007/s10071-022-01698-2 (PMC9950180; doi:10.1007/s10071-022-01698-2)
Supplement: Supplementary file 1 — Supplementary file1 (PDF 320 KB) [file 10071_2022_1698_MOESM1_ESM.pdf]

Paradoxical choice and the reinforcing value of information

Victor Ajuwon[1,*], Andrés Ojeda[1], Robin A. Murphy[2], Tiago Monteiro[1,3] and Alex Kacelnik[1,*]

[1]Department of Biology, University of Oxford, UK

[2]Department of Experimental Psychology, University of Oxford, UK

[3]Domestication Lab, Konrad Lorenz Institute of Ethology, Department of Interdisciplinary Life Sciences, University of Veterinary Medicine Vienna, Austria

*Correspondence and requests for materials should be addressed to V.A. (victor.ajuwon@biology.ox.ac.uk), or A.K. (alex.kacelnik@biology.ox.ac.uk).

Online resource 1 - Supplementary materials

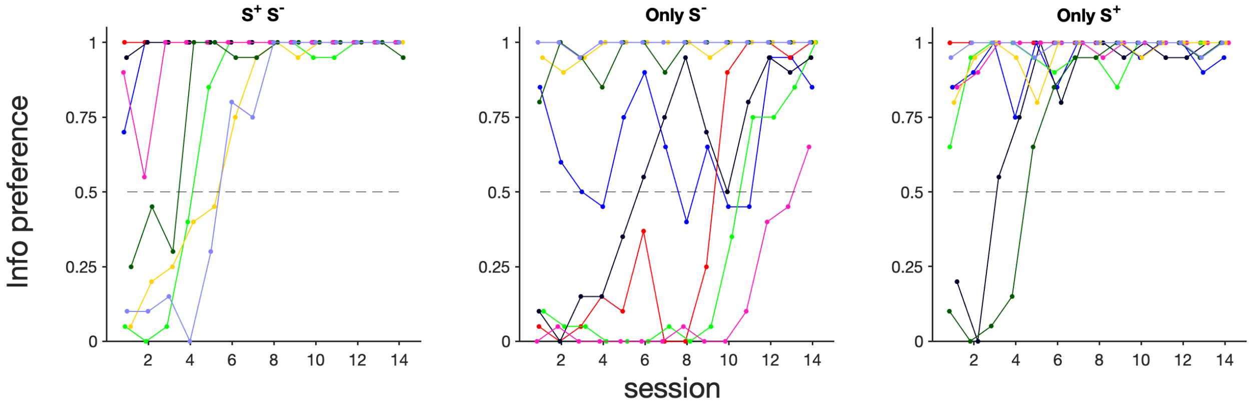

Figure S1. Individual preferences for the *Info* option. Proportion of observed preferences in the $S^{+}\_S^{-}$ (n = 8), *Only_*S^{-} (n = 8) and *Only_*S^{+} (n = 8) groups are shown. Each colour represents a different individual for each group.

Treatment

	$S^+\_S^-$	$Only\_S^-$	$Only\_S^+$
α	4.22 (4.06- 4.4)	10.78 (8.531- 13.03)	2.09 (-1.765 -5.95)
β	1.68 (1.47 - 1.89)	2.69 (1.70 - 3.70)	3.27 (1.201- 5.34)
γ	0.49 (0.46 - 0.51)	0.48 (0.44 - 0.53)	0.48 (0.01 - 0.95)

Table S1. Estimated parameter values from sigmoidal Gaussian curves fit to mean *Info* preference data in the main experiment. α gives the inflection point, β the slope at the inflection point, and γ the lower bound of the curves. The upper bound given by 1-λ was set at 1. 95% confidence bounds are given in brackets.
