# Supplementary material for: Paradoxical choice and the reinforcing value of information
Source: Anim Cogn. 2022 Oct 28;26(2):623–37. doi: 10.1007/s10071-022-01698-2 (PMC9950180; doi:10.1007/s10071-022-01698-2)
Supplement: Supplementary file 6 — Supplementary file6 (DOCX 182 KB) [file 10071_2022_1698_MOESM6_ESM.docx]

**Supplementary materials**


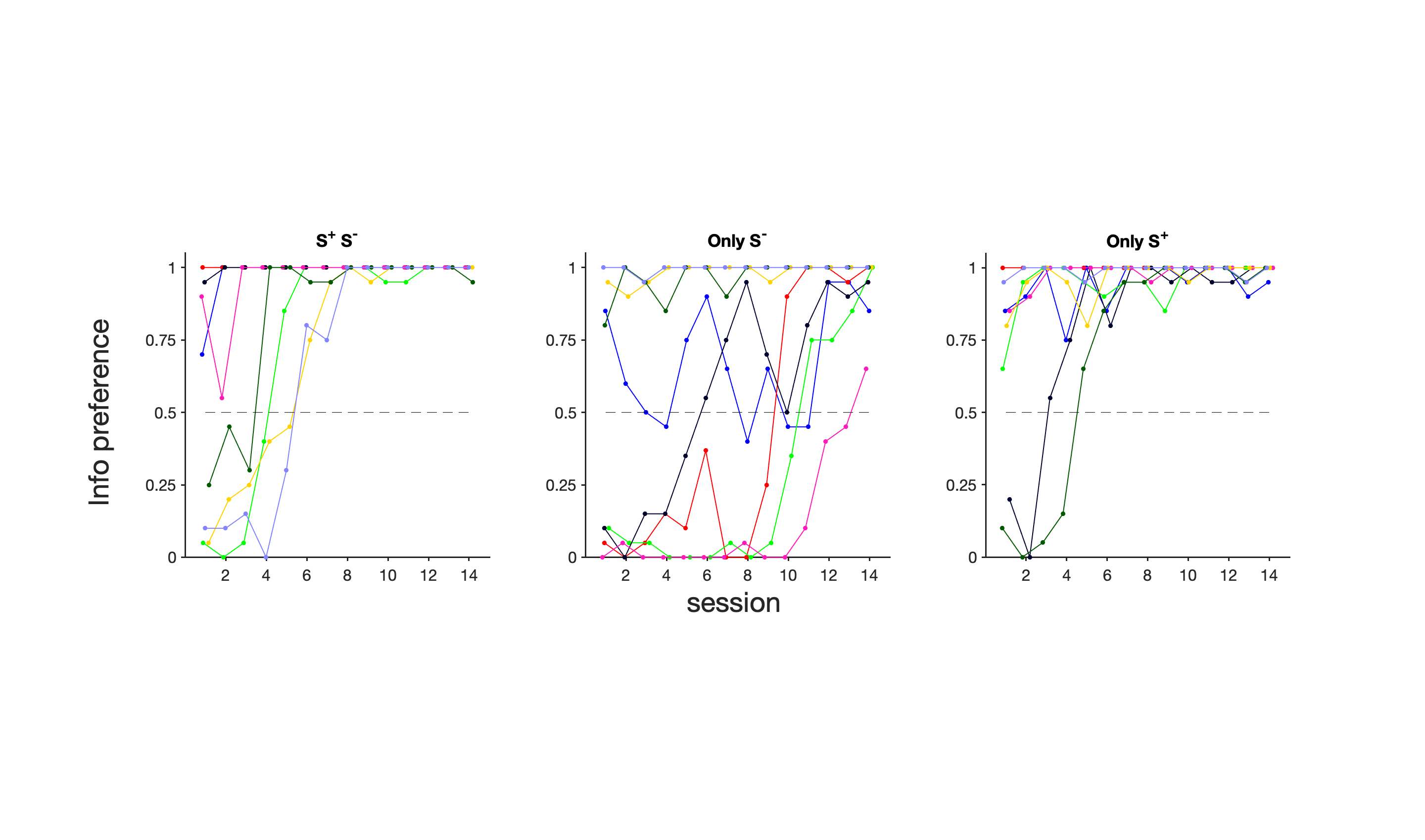


**Figure S1. Individual preferences for the *Info* option.** Proportion of observed preferences in the *S^+^_S^-^* (n = 8), *Only_S^-^* (n = 8) and *Only_S^+^* (n = 8) groups are shown. Each colour represents a different individual for each group.

| **Treatment** | | | |
| --- | --- | --- | --- |
|  | ***S^+^_S^-^*** | ***Only_S^-^*** | ***Only_S^+^*** |
| **α** | 4.22 (4.06- 4.4) | 10.78 (8.531- 13.03) | 2.09 (-1.765 -5.95) |
| **β** | 1.68 (1.47 - 1.89) | 2.69 (1.70 - 3.70) | 3.27 (1.201- 5.34) |
| **γ** | 0.49 (0.46 - 0.51) | 0.48 (0.44 - 0.53) | 0.48 (0.01 - 0.95) |

**Table S1. Estimated parameter values from sigmoidal Gaussian curves fit to mean *Info* preference data in the main experiment.** $\alpha$ gives the inflection point, $\beta$ the slope at the inflection point, and $\gamma$ the lower bound of the curves. The upper bound given by 1-λ was set at 1. 95% confidence bounds are given in brackets.
